# Genomic Patterns of Homozygosity and Genetic Diversity in the Rhenish German Draught Horse

**DOI:** 10.3390/genes16030327

**Published:** 2025-03-11

**Authors:** Johanna Sievers, Ottmar Distl

**Affiliations:** Institute of Animal Breeding and Genetics, University of Veterinary Medicine Hannover (Foundation), 30559 Hannover, Germany; johanna.sievers@tiho-hannover.de

**Keywords:** genomic inbreeding, ancestral inbreeding coefficients, runs of homozygosity, effective population size, ROH islands, selection signatures

## Abstract

Background/Objectives: The Rhenish German draught horse is an endangered German horse breed, originally used as working horse in agriculture. Therefore, the objective of this study was to evaluate the breed’s genetic diversity using pedigree and genomic data in order to analyze classical and ancestral pedigree-based inbreeding, runs of homozygosity, ROH islands, and consensus ROH. Methods: We studied the genome-wide genotype data of 675 Rhenish German draught horses and collated pedigree-based inbreeding coefficients for these horses. The final dataset contained 64,737 autosomal SNPs. Results: The average number of ROH per individual was 43.17 ± 9.459 with an average ROH length of 5.087 Mb ± 1.03 Mb. The average genomic inbreeding coefficient F_ROH_ was 0.099 ± 0.03, the pedigree-based classical inbreeding coefficient F_PED_ 0.016 ± 0.021, and ancestral inbreeding coefficients ranged from 0.03 (F_a_Kal_) to 0.51 (Ahc). Most ROH (55.85%) were classified into the length category of 2–4 Mb, and the minority (0.43%) into the length category of >32 Mb. The effective population size (N_e_) decreased in the last seven generations (~65 years) from 189.43 to 58.55. Consensus ROH shared by 45% of the horses were located on equine chromosomes 3 and 7, while ROH islands exceeding the 99th percentile threshold were identified on chromosomes 2, 3, 5, 7, 9, 10, and 11. These ROH islands contained genes associated with morphological development (*HOXB* cluster), fertility (*AURKC*, *NLRP5*, and *DLX3*), muscle growth, and skin physiology (*ZNF* gene cluster). Conclusions: This study highlights how important it is to monitor genetic diversity in endangered populations with genomic data. The results of this study will help to develop breeding strategies to ensure the conservation of the German Rhenish draught horse population and show whether favorable alleles from the overrepresented candidate genes within ROH were transmitted to the next generation.

## 1. Introduction

The Rhenish German draught horse is a German breed established in the late 19th century to meet the rising demands for heavy draught horses during agricultural intensification and industrialization [1]. By crossbreeding local working horses with imported Belgian draught horses, the foundation of the breed began, and the Rhenish studbook was established in 1892 to regulate its breeding [1,2]. Similar breeds, like the Mecklenburg draught horse and Saxon Thuringian draught horse, emerged in parallel, sharing the Belgian draught horse as a common ancestor [1]. The Rhenish German population grew rapidly, reaching its highest population size with 25,022 breeding horses in 1946 [2]. A severe decline in population size began in the late 1950s as mechanization in agriculture reduced the need for draught horses [2]. By 1974, the population in West Germany diminished to just 11 mares and two stallions [1,2]. Reunification of the East and West German populations in 1990, along with dedicated breeding efforts and diversification of the horse’s roles (e.g., forestry, leisure), helped to prevent extinction [1,2,3,4]. In 2004, all subpopulations of the Rhenish German draught horse were united into one studbook with all their pedigree data [3]. A total of 1137 active breeding animals, including 127 stallions and 1010 mares, are registered in the studbook of the Rhenish German draught horse [5]. The German Federal Office for Agriculture and Food (BLE) has included the Rhenish German draught horse as an observation population in the Red List of Endangered Livestock Breeds 2023 [6]. The BLE uses the effective population size (N_e_) based on the male and female breeding animals to determine the endangerment status of indigenous livestock breeds [6]. The N_e_ of the Rhenish German draught horse in the 2023 census by the BLE is 451, highlighting the need to monitor its genetic diversity to ensure the maintenance of this horse breed [6]. The drastic decline in population size in the 1950s to 1970s and subsequent recovery in the last 30 years, as well as selective breeding, may have affected the genetic diversity and inbreeding levels of the breed, thus warranting genomic analysis.

Before the genomic era, pedigree data were mostly used to estimate genetic diversity and inbreeding coefficients [7,8,9,10]. However, gaining access to pedigree information can be challenging, and ancestry data may be incomplete or entirely absent [11,12]. Furthermore, pedigree information can be unreliable, due to inaccurate documentation of ancestry [11,13]. This may influence the precision of the estimated results. The possibility of generating genomic data using single nucleotide polymorphisms (SNPs) has opened new opportunities for estimating genetic diversity and population structure in livestock species [14]. Previous studies have shown that genomic data offer greater accuracy than pedigree data and provide more possibilities to estimate the population structure and genetic diversity [11,14]. Nonetheless, pedigree analysis can complement genomic data to generate a comprehensive estimate of genetic diversity in a population [13].

A widely used method in equine studies to determine the degree of genomic inbreeding is the analysis of runs of homozygosity (ROH) [15,16,17,18,19,20]. ROH are defined as continuous regions of homozygosity within the DNA of an individual or population, occurring when identical haplotypes are inherited from common ancestor(s) of the parents, resulting in identical genetic sequences [12,21,22]. The classification of ROH into different length classes is an established method that has been utilized in several previous studies to obtain information on the temporal origin of inbreeding and to determine inbreeding events throughout breeding history [18,23,24,25]. Furthermore, the assessment of overlapping homozygous regions, known as ROH islands, which are shared by a significant proportion of individuals within a population, is frequently used to detect genomic regions associated with selection across various livestock breeds, including horses [12,26]. Among others, signatures of selection for coat color, body size [16,23,25,27,28,29], performance [16,30], disease resistance [25], immune system [29], and fertility [28,29,30,31] have been detected using ROH.

To date, no study has examined the genetic diversity of the endangered Rhenish German draught horse using genomic data. Therefore, the objective of this study was to evaluate the genetic diversity in a large sample of the Rhenish German draught horse population using genomic data. We estimated inbreeding coefficients based on both pedigree and genomic data and further analyzed ROH islands and consensus ROH to detect signatures of selection, as well as candidate genes for important breeding traits that were overrepresented in ROH.

## 2. Materials and Methods

### 2.1. Ethical Approval

The study was conducted in accordance with the guidelines of the Declaration of Helsinki, and approval was granted by the Institutional Review Board of the University of Veterinary Medicine Hannover (Foundation) and the state veterinary offices from the different German Federal States for North Rhine-Westphalia (registration number 81-02.05.40.19.083), Lower Saxony (registration number 33.8-42502-05-19A465), Thuringia (registration number 22-2684-04-TIH-20-101), Brandenburg (registration number 2347-A-19-1-2020), and Saxony (registration number 25-5131/521/20). European Union guidelines for the care and handling of animals and good veterinary practice were followed in the sampling and handling of horses.

### 2.2. Sample Collection and Genotype Data

The study included 675 Rhenish German draught horses, comprising 248 males and 427 females. EDTA-blood and hair root samples were collected between 2019 and 2023. We extended the number of samples used in our previous studies with 182 additional horses [32,33]. In this way, we were able to supplement the most recent birth cohorts with a larger number of horses and add horses with a birth year of 2022. Sampling took place across 96 different German horse farms in Brandenburg, Lower Saxony, Rhineland, Westphalia, and Thuringia. All horses were recorded by the studbook for Rhenish German draught horses of the respective regional horse breeding organization.

Pedigree information was provided by vit Verden (Vereinigte Informationssysteme Tierhaltung w.V.). This dataset included 20,242 records and ancestors born in the last 70 years. Horses sampled for the present study were born between 1988 and 2022. The mean number of equivalent generations (GE) for these 675 horses was 7.42 ± 1.12.

All 675 animals were genotyped with the Illumina GGP Equine Plus Beadchip (Neogen, Lincoln, NE, USA) containing 71.589 single nucleotide polymorphisms (SNPs). Only autosomal SNPs were used for all further analyses. All SNPs and animals with genotyping rates < 0.90 were excluded from the dataset. The remaining dataset contained 64,373 autosomal SNPs, and all animals reached a genotyping rate > 0.993. Quality control of SNP data was performed using PLINK v1.9 (www.cog-genomics.org/plink/1.9/, accessed on 2 January 2025), Complete Genomics, Mountain View, CA, USA [34].

### 2.3. Runs of Homozygosity and Fixation Index

Runs of homozygosity (ROH) were analyzed using the overlapping window approach implemented in PLINK v1.9 [34]. Pruning for minor allele frequency (MAF) and linkage disequilibrium (LD) was not performed in accordance with Meyermans et al. [35] and Lencz et al. [36]. This was justified, as our genotyping data showed a very low error rate and the SNPs were evenly distributed across the equine genome. Removing SNPs with a low MAF or deviating from the Hardy–Weinberg equilibrium should prevent the inclusion of SNPs due to genotyping errors. The SNPs on the genotyping platform used have proven to have good technical reproducibility and high polymorphism information content. Applying restrictions due to MAF would result in a loss of ROH, as SNPs that are polymorphic in other horse breeds would be removed from the analysis [35,36]. LD pruning should allow that ROH are detected with a relatively equal probability across the genome. Therefore, light to moderate LD pruning should only be performed when the SNPs are not uniformly distributed across the genome and thus, recombination distances between SNPs tend to be very different. The minimum number of SNPs for a ROH was calculated as proposed by Lencz et al. [36] and Purfield et al. [37] with a type I error rate (α) of 0.05, an average SNP heterozygosity of 0.273, and an average SNP density of 34.84 Kb per SNP. The following settings resulted for these data: a minimum SNP density of one SNP per 100 kb, a maximum gap length of 500 kb, a minimum length of homozygous segments of 2230 kb including 64 or more homozygous SNPs, and a window size of 15 SNPs. One heterozygous SNP genotype and one missing SNP was permitted. The total length of the autosomal chromosomes covered by SNPs was 2,241,761,617 bp. To visualize the ROH length distribution, the ROH segments were categorized into the following length segments: ≤4 Mb, >4–8 Mb, >8–16 Mb, >16–32 Mb and >32 Mb.

The inbreeding coefficients F_Hat1–3_ by Yang et al. were calculated using the genome-wide complex trait analysis (GCTA) [38]. F_Hat1_ estimates the variance explained by all autosomal SNPs, F_Hat2_ measures the excess of homozygosity, similar to the F_IS_ estimate, and F_Hat3_ partitions the genetic variance into each of the 31 autosomes [38].

The genomic inbreeding coefficient (*F_ROH_*) for each horse was estimated according to McQuillan et al. [39], including all ROH and ROH by length classes in Mb, comprisingF_ROH>4_, F_ROH>8_, F_ROH>16_, and F_ROH>32_, as well as F_ROH>6.739_, F_ROH-2–4_, F_ROH-4–8_, F_ROH-8–16_, and F_ROH-16–32_.

The fixation index *F_IS_* (excess of homozygosity) for each individual was calculated with the software SAS version 9.4 (Statistical Analysis System, Cary, NC, USA, 2024) [40].

### 2.4. Pedigree Based Inbreeding Coefficients

Pedigree data were used to calculate individual inbreeding coefficients (F_PED_) following Meuwissen and Luo [41] using PEDIG, version 5 [42] and the individual rate of inbreeding (ΔF_PED_) according to Gutiérrez, et al. [43] which is corrected for the pedigree depth of the individual animal.

The ancestral inbreeding coefficient (F_a_Bal_) by Ballou [44], the ancestral inbreeding (F_a_Kal_) and new inbreeding (F_New_) coefficients by Kalinowski et al. [45,46], and the ancestral history coefficient (Ahc) by Baumung et al. [47] were calculated based on the gene drop method implemented in GRAIN, version 2.2 [46,47]. These methods are described in more detail in previous studies [48,49].

### 2.5. Effective Population Size

The effective population size (N_e_) was estimated using PLINK (www.cog-genomics.org/plink/1.9/, accessed on 2 January 2025) version 1.9 [34] based on the LD, measured as the squared correlation (r^2^) between pairs of SNPs. The calculation of r^2^ values was based on SNP pairs, with a distance of 1 Kb to 50 Mb between each pair. The mean r^2^ value was estimated for distance bins of 10 Kb to 100 Kb. The N_e_ was calculated as $N_{e}=\frac{\left(1-r^{2}\right)}{\left(4cr^{2}\right)}$, with c being the recombination rate in Morgan units [50], which was approximated by the distance between two SNPs in units of 100 Mb (equal to 1 Morgan). The number of generations in the past was calculated as $\frac{1}{\left(2c\right)}$. The increase in inbreeding per generation was derived from $\Delta F=\frac{1}{2\;N_{e}}$ [43].

Another approach to calculate ΔF and N_e_ was based on F_ROH_, F_ROH>4_, F_ROH>8_, F_ROH>16_, and F_ROH>32_ and GE derived from the respective F_ROH_ [43,46]:

$$\Delta F_{ROH-i}=1-\sqrt[{GE-ROH_{i}-1}]{(1-F_{ROH-i}})\;and{\;N}_{e-ROH-i}=1/2\Delta F_{ROH-i}$$

with F*_ROH-i_* = F_ROH_, F_ROH>4_, F_ROH>8_, F_ROH>16_, and F_ROH>32_. The parameter GE-ROH_i_ corresponded to 15.6691, 12.5, 6.25, 3.125, and 1.5625 generations for F_ROH_, F_ROH>4_, F_ROH>8_, F_ROH>16_, and F_ROH>32_.

### 2.6. ROH Islands, Consensus ROH and Gene Onotolgy Enrichment

ROH islands were defined as regions that exceeded the 99th percentile of the homozygosity distribution. In addition, we determined consensus ROH, which were shared by 20%, 30%, 40%, and 45% of the horses. The Ensemble genome assembly release 112 of EquCab3 [51] was used to retrieve gene annotations for ROH islands and consensus ROH regions. PANTHER v17.0 (Protein Analysis Through Evolutionary Relationships), developed by the Division of Bioinformatics, Department of Preventive Medicine, Keck School of Medicine of USC, University of Southern California, Los Angeles, CA, USA [52] was employed to elucidate the molecular functions and biological processes for these genes. Furthermore, we ran overrepresentation tests [53]. In addition, previously reported QTLs within the identified ROH islands were retrieved using the Animal QTLdb [54,55] (https://www.animalgenome.org/cgi-bin/QTLdb/BT/index, accessed on 18 January 2025).

## 3. Results

### 3.1. ROH and Inbreeding Coefficients

The average number of ROH per horse was 43.17 ± 9.459, with an average ROH length of 5.087 Mb ± 1.03 Mb and a combined length of 222.487 ± 67.804 Mb (Table 1).

The average F_ROH_ and F_IS_ were 0.099 ± 0.030 and 0.006 ± 0.069, respectively (Table 2). All pedigree-based inbreeding coefficients were lower than F_ROH_. F_ROH>8_ resulted in 2.19-fold higher inbreeding than F_PED_. F_ROH>6.739_, which corresponds to 7.42 generations, reached an estimate of 0.041 ± 0.026, which is 2.59-fold higher than F_PED_. However, when comparing median values, F_PED_ and F_ROH>6.739_ were very similar. F_HAT3_ showed the highest estimate with 0.046, and F_HAT2_ the lowest with 0.025.

F_PED_ and F_ROH_ were moderately associated with a correlation coefficient of 0.591, whereas ΔF_PED_ reached a higher correlation coefficient of 0.719 with F_ROH_ (Table 3). Generally, ΔF_PED_ showed a higher similarity with ROH-based inbreeding coefficients than F_PED_. The correlation coefficients between F_PED_ (ΔF_PED_) and F_ROH>8_ were highest among the different F_ROH_ with an estimate of 0.654 (0.783). The lowest correlation coefficient was found between F_PED_ and F_ROH-2–4_ with an estimate of 0.029. Similarly, the corresponding correlation coefficient for ΔF_PED_ with F_ROH-2–4_ was 0.072. F_ROH_ showed the highest correlation of 0.962 with F_ROH>4_ and the lowest of 0.386 with F_ROH-2–4_.

**Figure 1:**
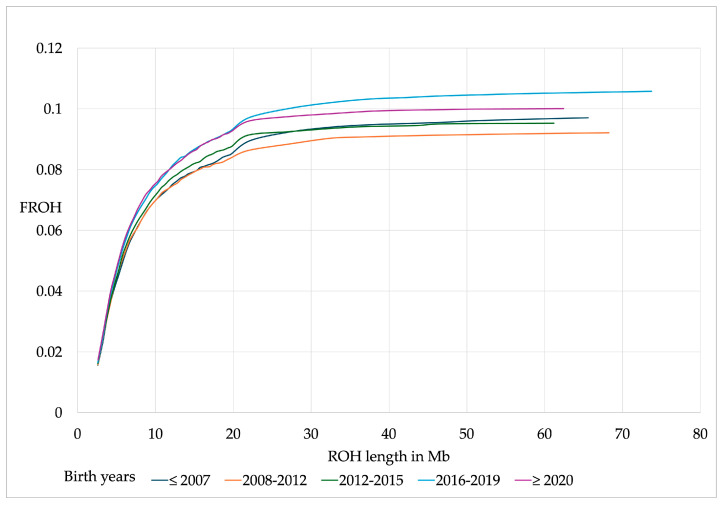
Cumulative F_ROH_ by birth cohorts.

The cumulative distribution of F_ROH_ by ROH lengths showed a steep increase up to a length of 20 Mb in animals from all birth years (Figure 1). Horses of earlier birth cohorts (≤2007–2015) exhibited a slightly lower increase than horses of later birth cohorts (2016 to ≥2020) (Figure 1). Differences for F_ROH_, average and total ROH length, and number of ROH per horse were not significantly different between birth year cohorts.

The majority of ROH were classified into the length category of 2–4 Mb with 16,276 ROH, representing 55.85% of all ROH (Table 4). The lowest number of ROH, with 124 ROH (0.43%), were in the length category of >32 Mb.

**Figure 2:**
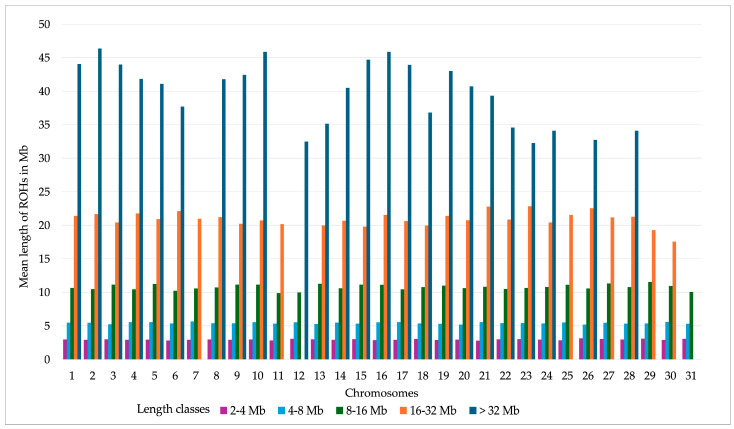
Mean length of runs of homozygosity (ROH) across autosomal chromosomes, categorized into different length classes, in Rhenish German draught horses (n = 675).

The distribution of the average lengths of ROH by the different length classes across each autosomal chromosome is given in Figure 2. The greatest variability in the average ROH length was seen for the class > 32 Mb. On chromosomes 7, 11, 25, 27, 29, 30, and 31, there were no ROH for the > 32 Mb length class. The combined length of ROH per chromosome was proportional to the length of the respective autosome (Figure S1).

**Figure 3:**
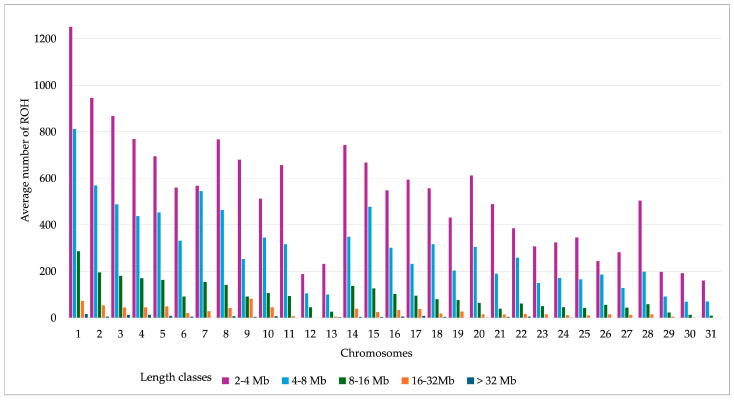
Average number of runs of homozygosity (ROH) across autosomal chromosomes, categorized into different length classes in Rhenish German draught horses (n = 675).

The average number of ROH by autosomal chromosomes, categorized into different length classes, is shown in Figure 3. The length class 2–4 Mb showed the highest average number of ROH on chromosome 1. The length category > 32 Mb harbored the lowest average number of ROH on each autosome.

### 3.2. Effective Population Size

**Figure 4:**
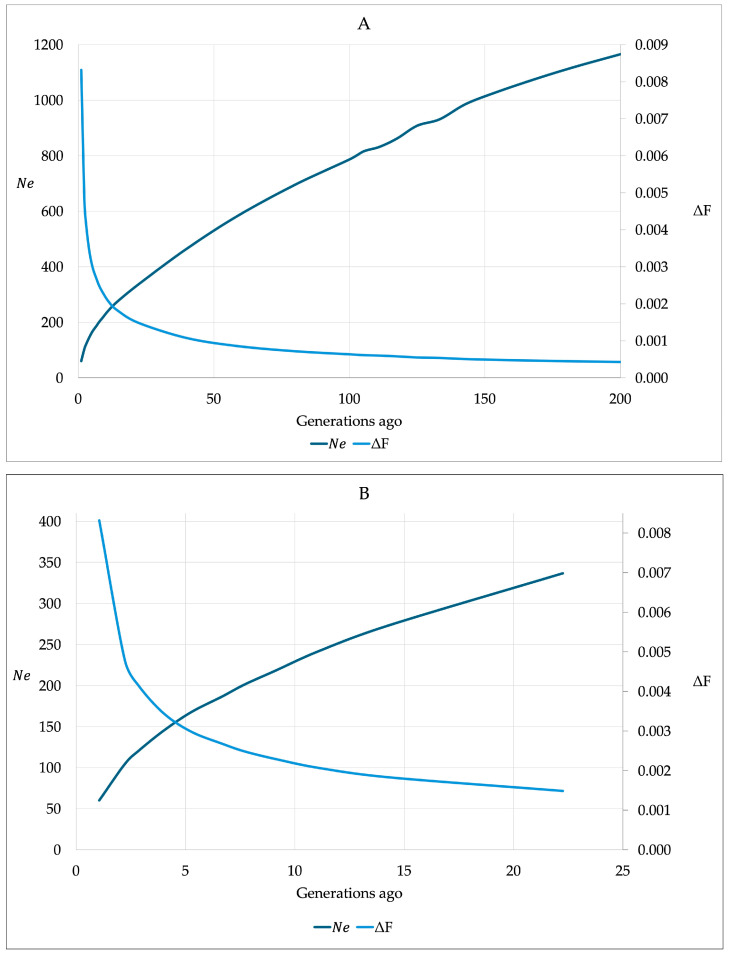
Change in effective population size (N_e_) and increase in inbreeding per generation (∆F) in the last (**A**) 200 generations and (**B**) 20 generations based on Rhenish German draught horses born in 1988–2022 and linkage disequilibrium estimates from pairs of SNPs.

The effective population size N_e_ was calculated using r^2^ values between pairs of SNPs to show the trends for N_e_ and ΔF for the last 200 generations (Figure 4). It was found that 200 generations ago, N_e_ reached an estimate of 1184.61. The development of N_e_ over the generations demonstrated a long-lasting downward trend, which has accelerated rapidly in the last five generations. The decline in N_e_ from generation 20 to 10 was from N_e_ = 326.99 to 219.80 (−10.72 per generation) and in generations 5, 4, 3, 2, and 1, N_e_ = 163.04, 144.82, 124.40, 98.74, and 58.55 (−20.90 per generation), respectively. The development of ΔF over generations was inverse to N_e_.

We also estimated ΔF_ROH_ and N_e-ROH_ for F_ROH_, F_ROH>4_, F_ROH>8_, F_ROH>16_, and F_ROH>32_ from the overall means of ROH from all horses. The resulting estimates for ΔF_ROH_ were 0.0071, 0.006060, 0.006746, 0.006896, and 0.00608, with corresponding estimates for N_e-ROH_ of 70.42, 82.51, 74.12, 72.51, and 82.21, respectively.

**Figure 5:**
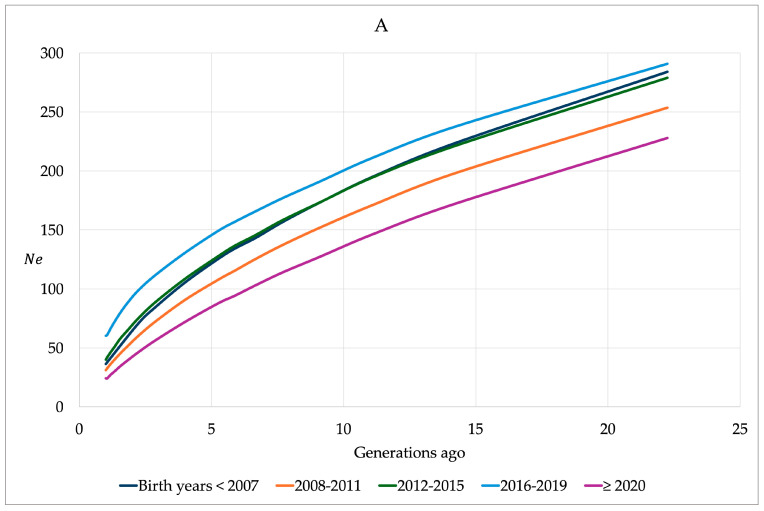

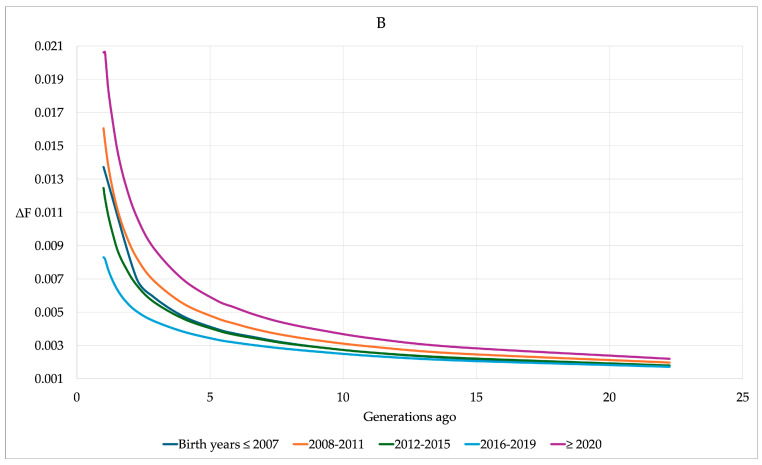
Effective population size (N_e_) (**A**) and increase in inbreeding per generation (∆F) (**B**) in Rhenish German draught horse for the last 20 generations using linkage disequilibrium.

Analyses for N_e_ and ΔF based on LD between pairs of SNPs were also performed according to the birth year cohorts (Figure 5). The decline in N_e_ was similar in all five birth year cohorts. Horses with birth years ≥ 2020 showed the lowest values for N_e_, and horses with birth years 2016–2019 the highest in the last 20 generations. The trends for N_e_ and ΔF by birth year classes over 200 years revealed the highest N_e_ for the birth years ≤ 2007 and 2012–2019 and the lowest N_e_ for the birth years 2008–2011 and ≥2020 (Figure S2).

### 3.3. Consensus ROH and ROH Islands

The 31 consensus ROH shared by 25% and 30% of the horses were identically distributed and located on 14 equine chromosomes (ECA 1–5, 7–11, 20–22 and 28) (Table S2a,b). Consensus ROH shared by 40% and 45% of the horses were located on ECA 3, 5, 7, and 11, as well as on ECA 3 and 7 (Table S2c,d). The longest segment for a 30% consensus ROH was located on chromosome 10 between 23.15 and 29.59 Mb, containing 186 SNPs. The 45% consensus ROH on ECA 3 and 7 contained 51 and 55 SNPs, respectively.

ROH islands, exceeding the 99th percentile threshold, were identified on ECA 2, 3, 5, 7, and 9–11 (Table 5 and Table S3). The number of identified genes ranged from 6 on ECA 3 to 99 on ECA 10.

The PANTHER gene ontology enrichment analysis for ROH islands is shown in Table S3. We found a statistically significant overrepresentation on ECA 9 in the category “PANTHER Go-Slim Biological Process”, on ECA 10 for the categories “PANTHER Go-Slim Biological Process”, “PANTHER Go-Slim Molecular Function”, and “PANTHER Protein Class”, and on ECA 11 for the categories “PANTHER Go-Slim Biological Process” and “PANTHER Protein Class”. The ECA 11 ROH island contained genes of the homeobox B cluster including *HOXB1*, *HOXB2*, *HOXB3*, *HOXB4*, *HOXB5*, *HOXB8*, *HOXB9*, and *HOXB13*. On ECA 10, the *aurora kinase C* gene (*AURKC*) was identified as significantly overrepresented. Furthermore, we identified many genes from the zinc finger protein group (ZNF) (*ZNF17*, *ZNF71*, *ZNF264*, *ZNF304*, *ZNF444*, *ZNF470*, *ZNF471*, *ZNF543*, *ZNF550*, *ZNF581*, *ZNF582*, *ZNF583*, *ZNF667*, *ZNF772*, *ZNF773*, *ZNF784*, *ZNF787*, *ZNF865*) on ECA 10.

QTLs located within the ROH islands of the 99th percentile and 25% consensus ROH are summarized in Table S4a and Table S4b, respectively. We found QTLs on ECA 2, 3, 5, 9, and 11, including QTLs associated with female fertility, height at withers, overall body size, insect bite hypersensitivity, and hair density.

## 4. Discussion

This is the first study on the detection of ROH and ROH islands and which searches for overrepresented genes within these homozygous DNA segments in the Rhenish German draught horse using genome-wide data. The mean and median F_ROH_ for the whole population were estimated as 9.9% and 9.5%. Restricting the ROH segments to a size of more than 6.739 Mb, which approximately corresponds to ROH that arose within the last 7.42 generations, revealed a mean and median for F_ROH>6.739_ of 4.1% and 3.6%, respectively. Compared with F_PED_ based on pedigree data with a GE of 7.42, the medians were in the same range for both F_ROH>6.739_ and F_PED_. However, for a majority of animals, inbreeding is underestimated when using F_PED_. ΔF_PED_ showed a higher correlation to F_ROH_ than F_PED_, and the difference between its mean value and that of F_ROH>6.739_ is smaller compared with F_PED_. In summary, the SNP data captured more information from generations ago, and thus enabled us to characterize the genetic diversity and inbreeding more precisely than with pedigree data alone. Other studies in draught horse breeds have also found higher inbreeding with genomic data compared with pedigree data. Belgian draught horses, genealogically related to the Rhenish German draught horse, had a similar F_ROH_ of 10.1% (n = 23) [20]. Noriker and Norik of Muran discovered similar inbreeding levels as well, with 10% and 11%, respectively [17,27]. Polish Coldblood horses showed a lower inbreeding estimate of 6.1%, whereas Italian heavy draught and Friesian horses exhibited higher inbreeding estimates of 15.36% and 22.3% [18,20,56].

Comparing ROH analyses from different studies is challenging because parameters used to determine ROH do not always adhere to the recommendations [35,36]. Mancin [18] and Polak et al. [56] pruned for MAF and applied the Hardy–Weinberg equilibrium (HWE), whereas Grilz-Seger and Druml [27] and Kasarda et al. [17] only pruned for MAF. Schurink et al. did not prune for MAF or apply HWE, but pruned for LD [20]. Pruning for MAF and LD may result in missing ROH and therefore influence the assessment of inbreeding estimates [35]. Particularly, when the study does not include a larger number of animals from different breeds, pruning for MAF and LD may lead to less ROH for the analysis.

The classification of ROH segments into different length categories can provide an insight into the timing of inbreeding events. Longer ROH are considered to be a sign of recent inbreeding, whereas shorter ROH are considered to be a sign of more ancient inbreeding [24]. In the Rhenish German draught horse, the majority of ROH can be classified into length segments between 2–4 Mb (F_ROH-2–4_), and the minority is grouped into length segments of >32 Mb (F_ROH>32_). Assuming that 1 cM is 1 Mb, a mean ROH length of approximately 2.95 Mb represents inbreeding events occurring about 17 generations ago. Using a generation interval estimate of 10 years, we date these inbreeding events to around 170 years ago, or approximately 1844. This date is approximately 50 years before the establishment of the Rhenish studbook in 1892 [2]. The breed originated from crossbreeding of local working horses with imported Belgian draught horses [1,2]. This study suggests that in the early stages of Rhenish German draught horse breeding, closer related horses were more likely to be bred together, possibly to accelerate the achievement of desired breed characteristics. Additionally, the lack of an established studbook could have led to unregulated breeding strategies that did not consistently take into account genetic relationships. Additionally, the length class of 4–8 Mb (F_ROH-4–8_), with a mean ROH length of 5.44 Mb, is frequently observed in this population, indicating inbreeding events estimated to have occurred between 1900 and 1962. During this period, the number of Rhenish German draught horses declined drastically due to the reduced demand for heavy draft horses after the 1950s, leaving only a limited number of broodmares and stallions [1,2]. To prevent extinction, some level of inbreeding was probably inevitable. Even though ROH segments of >32 Mb exist at a low frequency, in the most recent generations, inbreeding rate per generation increased, and current breeding strategies are not adapted to effectively reduce the risk of inbreeding.

The inbreeding coefficient F_PED_, based on pedigree information, was 1.6%, closely matching a previous study by Biedermann et al., which estimated an inbreeding coefficient of 1.7% in 2002 [7]. Similarly, Aberle et al. calculated inbreeding coefficients for several German draught horses, including the Rhenish German draught horse, at 1.53%, which aligns well with both our findings and Biedermann’s previous study [1,7]. In comparison, subpopulations of the Rhenish German draught horse, specifically the Saxon Thuringa Coldblood and Mecklenburg Coldblood, showed higher inbreeding coefficients of 2.13% and 2.61% [1]. The accuracy of inbreeding coefficients based on pedigree data is largely dependent on the completeness and depth of the pedigrees used [11,12,13]. In our study, pedigrees were available for 7.42 GE, which may limit the detection of inbreeding events beyond this generational depth. As previously mentioned, a considerable part of inbreeding in the Rhenish German draught horse population likely occurred in ancient times [24]. We assume that the limited pedigree depth prevents detection of these ancient inbreeding events in pedigree-based analysis, and therefore may explain the discrepancy between F_PED_ and F_ROH_. This assumption is supported by similar findings in previous studies [56,57]. Furthermore, inbreeding coefficients estimated from ROH length classes > 8 Mb (8–16 Mb, 16–32 Mb, >16 Mb, and >32 Mb) are more alike to pedigree-based inbreeding coefficients, reinforcing our hypothesis, because longer ROH represent more recent inbreeding [24]. These findings underline the importance of genomic data for comprehensive monitoring of genetic diversity in small populations. Incorporating genomic data into routine monitoring can provide better information on breeding strategies, helping to manage genetic diversity effectively, reduce inbreeding, and ensure the long-term health of the population.

The effective population size (N_e_) estimated based on linkage disequilibrium (LD) for the last generation of the Rhenish German draught horse was 58.55, which differs from previous estimates. For instance, Druml et al. estimated the N_e_ of the Rhenish German draught horse based on LD as 46.1, with a sample size of 46 horses [58]. In a subsequent analysis, Druml et al. grouped the Altmaerkisch draught horse, Mecklenburg Coldblood, Saxon Thuringa Coldblood, and the Rhenish German draught horse into a hypothetical population (n = 109), resulting in an higher estimate for N_e_ of 131.8 [58]. The study of Aberle et al. estimated N_e_ as 300 based on pedigree data [1]. In the present study, N_e_ derived from ΔF_PED_ reached 161, but N_e-ROH_ reached only 70.42. Other draught horse breeds such as the Italian Heavy draught horse, Noriker, and South German Coldblood have been found to have an effective population size of 100, 157.4, and 413 [1,10,18]. Only the Schleswig draught horse had a comparable N_e_ value of 89 [1]. The methodologies also varied. Mancin et al. estimated N_e_ using LD, Aberle et al. relied on pedigree analysis, and Druml et al. applied both methods [1,10,18,58]. Direct comparison of *N_e_* estimates across studies is challenging due to variations in methods and sample sizes. However, the estimated N_e_ values of the Rhenish German draught horse remain low, indicating reduced genetic diversity [59]. The present study further revealed varying N_e_ values across different birth cohorts. The cohort born between 2016 and 2019 exhibited the highest N_e_, suggesting higher genetic diversity in these cohorts. In contrast, the ≥2020 cohort showed the lowest N_e_ across all generations, indicating a loss of genetic diversity during these years. Further research is required to determine the exact causes of these discrepancies.

The PANTHER overrepresentation tests identified overrepresented genes on ECA 11, which includes the homeobox B (*HOXB*) gene cluster. The *HOXB* cluster plays a fundamental role in animal morphological diversity, controlling the body’s axial morphology along the anteroposterior axis and contributing to the embryonic development of the skeletal system [60]. This cluster has also been found in ROH islands of other horse breeds, including the Noriker, Lipizzan, Posavina, Gidran, and Soviet Heavy draught horses, as well as German sport horse breeds (Hanoverian, Holstein, Oldenburg, and Trakehner) [23,27,31,61,62].

Further, *AURKC* was identified in the ECA 10 ROH, which is associated with fertility in mares and stallions [63,64]. *ZNF304*, *ZNF543*, and *ZNF773* were also identified within an ROH island of Polish draught horse breeds (Sokolski, Sztumski) and the Hucul horse [65]. On ECA 9, we identified the *PRKDC* gene, which harbors the mutation for Severe Combined Immunodeficiency (SCID) [66]. Additionally, we found the *SP6* gene overrepresented, which has been identified as a QTL for hair density, and is associated with curly coat in Bashkir Curly Horses and Missouri Foxtrotters [67]. Nazari et al. found the same QTL for hair density in Kurdish horse breeds, and suggests that selection for thick skin with a dense coat may provide an advantage in cold winters and serve as protection against insect bites [68]. This hypothesis might well align with the selection towards robustness in the Rhenish German draught horse. Further, limb hair characteristics and skinfold thickness have been identified to be correlated with the prevalence of chronic progressive lymphedema (CPL) [33,69,70], showing skinfolds and fibrosis [71,72,73]. We may hypothesize that the *SP6* gene may contribute to the development of skinfolds and increased hair density in pasterns and thus to CPL. However, further research is needed to confirm a potential functional role of *SP6* in the Rhenish German draught horse population.

On ECA 11, we also identified the *NFE2L1* gene, which was also observed in the Soviet heavy draught horse in a ROH hotspot [61]. *NFE2L1* is associated with several metabolic processes, including glucose, lipid, and protein metabolism [74]. Dementieva et al. suggested that heavy draught horses typically have a significant amount of brown fat, which may relate to their metabolic adaptations [61].

The *DLX3* gene on ECA 11 is part of the homeobox family and is involved in placental hormone regulation and cytotrophoblast differentiation in humans and placental development in mice [75]. It has also been detected in equine trophoblasts [76]. *AURKC* on ECA 10 was also found to be overrepresented. This gene is associated with fertility in mares, and with spermatogenesis in stallions [63,64]. Aurora protein kinases are involved in the regulation of many different processes during cell division, including the control of centrosome and spindle function, kinetochore-microtubule interactions, and cytokinesis [77,78,79]. In mares it is hypothesized that a reduction in AURKC kinase activity may contribute to aging-induced spindle instability, potentially leading to aneuploidy in oocytes [63]. In humans, failures in chromosome segregation at meiosis result in aneuploidy, which is a major cause of miscarriages and birth defects [80]. However, further research is needed to confirm the role of *AURKC* in the fertility of Rhenish German draught horse.

On ECA11, we identified a QTL associated with overall body size, also found in the Noriker [16,81,82]. Additionally, on ECA 2, 3, 5, 9, and 11, we determined 19 different QTLs associated with withers height, indicating a selection towards body size [83,84].

Other overrepresented genes, found on ECA 10, are part of the *NLR* family pyrin domain, specifically *NLRP4*, *5*, and *13*. Genes of the *NLR* family play an important role in the mammalian reproductive and immune system [85]. In mice, knocking out *NLRP5* leads to infertility due to arrest of preimplantation embryos, highlighting its importance in reproduction [86,87]. In cattle, *NLRP5* has been identified as a QTL associated with reproductive traits [87,88,89]. Further, *NLRP5* is involved in porcine preimplantation and early embryogenesis [87]. To date, the impact of *NLRP5* on equine reproduction remains unexplored. Further, on ECA 2, we identified a QTL associated with female fertility [90].

On ECA 10, we identified several genes from the zinc finger protein group. Zinc finger proteins (ZNFs) play key roles in regulating various cellular processes [91]. They are involved in transcriptional regulation, ubiquitin-mediated protein degradation, signal transduction, actin targeting, DNA repair, cell migration, and numerous additional cellular functions [91]. However, the ultimate function and purpose of most zinc-finger genes is unknown [92]. The genes *ZNF304*, *ZNF543*, and *ZNF773* were also identified in an ROH island of Polish draught horse breeds (Sokolski, Sztumski) and the Hucul horse [65]. Due to the role of ZNF*s* in muscle growth and differentiation, Szmatoła et al. suggested that *ZNF* genes may be linked to selection towards strength and muscle mass, particularly in draught horse breeds [65,91]. The overrepresentation of *ZNF* genes on ECA 10 may suggest a selection signature for strength and endurance, aligning with the historical use of the Rhenish German draught horse as a draught breed in agriculture. Additionally, the ZNF protein family plays an important role in skin physiology, including cell proliferation, differentiation, and apoptosis, thereby maintaining tissue homeostasis [91]. Further *ZNF* genes are involved in keratinocyte differentiation [91]. The involvement of ZNF proteins in keratinocyte differentiation may be associated with CPL, which is a common disease in the Rhenish German draught horse [32,69,71]. Given the role of ZNF proteins in skin cell differentiation and physiology, the association between *ZNF* overrepresentation and CPL prevalence in draught breeds warrants further exploration.

On ECA 9, we identified the *PRKDC* gene, which harbors the mutation for Severe Combined Immunodeficiency (SCID), found in Arabian horses but not in the Rhenish German draught horse [66].

## 5. Conclusions

The results of this study demonstrate a long-lasting downward trend in genetic diversity, with inbreeding events occurring in the late 19th century, prior to the establishment of the stud book. As a consequence of this decline in genetic diversity, the effective population size of the Rhenish German draught horse has reduced to 60–70 in the most recent generations. While the identified ROH islands did not indicate a sharp focus of selection towards one breeding objective, several islands were associated with traits related to reproduction and morphological traits. Overall, our study emphasizes the importance of using genomic data to monitor the genetic diversity of small populations and to develop strategies for maintaining long-term conservation efforts for the Rhenish German draught horse. The results of this study may also be beneficial for further research to analyze these loci within ROH for inbreeding depression or possible positive effects on performance and health.

## Figures and Tables

**Table 1:** Mean, standard deviation (SD), minimum (Min), and maximum (Max) of the average number of ROH, average ROH length, and combined length of ROH in all genotyped Rhenish German draught horses (n = 675).

ROH Items	Mean	SD	95% CI	75% CI	Min	Max
Average number of ROH	43.17	9.459	32–56	38–48	0	164
Average ROH length (Mb)	5.087	1.030	3.954–6.804	4.500–5.480	0	10.858
Combined ROH length (Mb)	222.487	67.804	139.036–337.123	179.984–257.994	0	630.724

**Table 2:** Means, medians, modes, standard deviations (SD), and 95% and 75% confidence intervals (CI) of pedigree and genome-based inbreeding coefficients for all genotyped Rhenish German draught horses (n = 675).

Inbreeding Coefficients	Mean	SD	Median	Mode	95% CI	75% CI
F_PED_	0.016	0.021	0.038	0.000	0.000–0.058	0.000–0.022
ΔF_PED_	0.0031	0.0035	0.0020	0.000	0.000–0.0101	0.0009–0.0040
F_a_Bal_	0.047	0.036	0.049	0.000	0.000–0.108	0.004–0.074
Ahc	0.051	0.040	0.052	0.000	0.000–0.147	0.000–0.079
F_a_Kal_	0.003	0.006	0.003	0.000	0.000–0.084	0.000–0.015
F_New_	0.012	0.018	0.012	0.000	0.001–0.051	0.019–0.034
F_IS_	0.006	0.069	0.005	−0.028	−0.056–0.126	−0.013–0.025
F_HAT1_	0.029	2.128	−0.126	−0.149	−0.243–−0.846	−0.172–−0.064
F_HAT2_	0.025	0.635	0.094	−0.149	−0.107–0.206	0.041–0.134
F_HAT3_	0.046	0.935	−0.001	0.000	−0.027–0.048	−0.012–0.016
F_HOM_	0.727	0.019	0.726	0.722	0.714–0.742	0.721–0.732
F_ROH_	0.099	0.030	0.095	0.000	0.062–0.150	0.080–0.115
F_ROH>4_	0.068	0.028	0.063	0.000	0.032–0.119	0.050–0.081
F_ROH>6.739_	0.041	0.026	0.036	0.000	0.011–0.088	0.025–0.053
F_ROH>8_	0.035	0.024	0.030	0.000	0.006–0.077	0.019–0.046
F_ROH>16_	0.015	0.019	0.009	0.000	0.000–0.048	0.000–0.020
F_ROH>32_	0.003	0.010	0.000	0.000	0.000–0.021	0.000–0.000
F_ROH-2–4_	0.032	0.008	0.032	0.000	0.016–0.043	0.027–0.036
F_ROH-4–8_	0.033	0.010	0.032	0.000	0.017–0.049	0.026–0.039
F_ROH-8–16_	0.020	0.011	0.019	0.000	0.004–0.042	0.013–0.028
F_ROH-16–32_	0.011	0.013	0.009	0.000	0.000–0.035	0.000–0.018

**Table 3:** Pearson correlation coefficients for selected pairs of inbreeding coefficients for the Rhenish German draught horse (n = 675). Pearson correlation coefficients for all pairs of inbreeding coefficients are displayed in Supplementary Table S1.

	ΔF_PED_	F_IS_	F_ROH_	F_ROH>4_	F_ROH>8_	F_ROH>16_	F_ROH>32_	F_ROH-2–4_	F_ROH-4–8_	F_ROH-8–16_	F_ROH-16–32_
F_PED_	0.981	0.315	0.591	0.624	0.654	0.646	0.565	0.029	0.144	0.353	0.469
ΔF_PED_		0.359	0.719	0.756	0.783	0.763	0.602	0.072	0.183	0.436	0.607
F_IS_			0.695	0.593	0.468	0.382	0.248	0.526	0.501	0.384	0.346
F_ROH_				0.962	0.871	0.759	0.513	0.386	0.551	0.634	0.672
F_ROH>4_					0.931	0.822	0.561	0.120	0.510	0.662	0.723
F_ROH>8_						0.898	0.653	0.019	0.162	0.686	0.758
F_ROH>16_							0.727	−0.016	0.107	0.295	0.845
F_ROH>32_								−0.029	−0.023	0.216	0.247
F_ROH-2–4_									0.282	0.067	0.001
F_ROH-4–8_										0.175	0.169
F_ROH-8–16_											0.248

**Table 4:** Number and average ROH lengths (Mb) with their standard deviations (SD), minima (Min), and maxima (Max) by length classes of Rhenish German draught horses (n = 675).

Length Classes in Mb	Number of ROH	Percent	Mean Length	SD	Min	Max
2–4	16,276	55.85	2.950	0.489	2.230	4.000
4–8	9074	31.14	5.437	1.074	4.000	7.998
8–16	2862	9.82	10.740	2.154	8.002	15.999
16–32	804	2.76	21.026	3.984	16.007	31.996
>32	124	0.43	41.802	10.350	32.127	84.829

**Table 5:** ROH islands with their chromosomal location (ECA), start and end position in bp, and number of included SNPs and genes, defined as the 99th percentile in Rhenish German draught horses (n = 675).

ECA	Start Position (bp)	End Position (bp)	Number of SNPs	Number of Genes
2	90,811,943	93,383,936	75	16
3	103,264,491	106,074,186	70	6
5	48,051,957	53,299,414	274	48
7	43,493,098	49,256,338	66	71
9	34,324,099	38,862,009	63	13
10	24,466,886	26,947,180	127	71
11	23,118,264	29,557,882	175	99

## Data Availability

Restrictions apply to the availability of these data. Data were obtained from German horse owners, breeders and the Rhenish, Westphalian, Brandenburg-Anhalt, Thuringian, and Lower Saxony breeder associations, and are available from the authors at a reasonable request and with the permission of the horse owners.

## Supplementary Materials

The following supporting information can be downloaded at: https://www.mdpi.com/article/10.3390/genes16030327/s1, Figure S1: Effective population size (N_e_) (A) and increase in inbreeding (∆F) (B) in Rhenish German draught horse for 200 generations, adapted from linkage disequilibrium and classified into different birth classes; Figure S2: Length of runs of homozygosity (ROH) across autosomal chromosomes in Rhenish German draught horses (n = 675); Table S1: Pearson correlation coefficients for all pairs of inbreeding coefficients; Table S2a: Consensus ROH shared by at least 25% of the horses; Table S2b: Consensus ROH shared by at least 30% of the horses; Table S2c: Consensus ROH shared by at least 40% of the horses; Table S2d: Consensus ROH shared by at least 45% of the horses; Table S3: PANTHER gene annotation and enrichment analysis of ROH islands; Table S4a: Quantitative trait loci that are located within the ROH islands of the 99th percentile threshold, retrieved from the public Animal QTLdb database [55,56]; Table S4b: Quantitative trait loci that are located within the 25% consensus ROH, retrieved from the public Animal QTLdb database [55,56].
