# Supplementary figures and images for: Genomic Patterns of Homozygosity and Genetic Diversity in the Rhenish German Draught Horse
Source: Genes (Basel). 2025 Mar 11;16(3):327. doi: 10.3390/genes16030327 (PMC11942601; doi:10.3390/genes16030327)

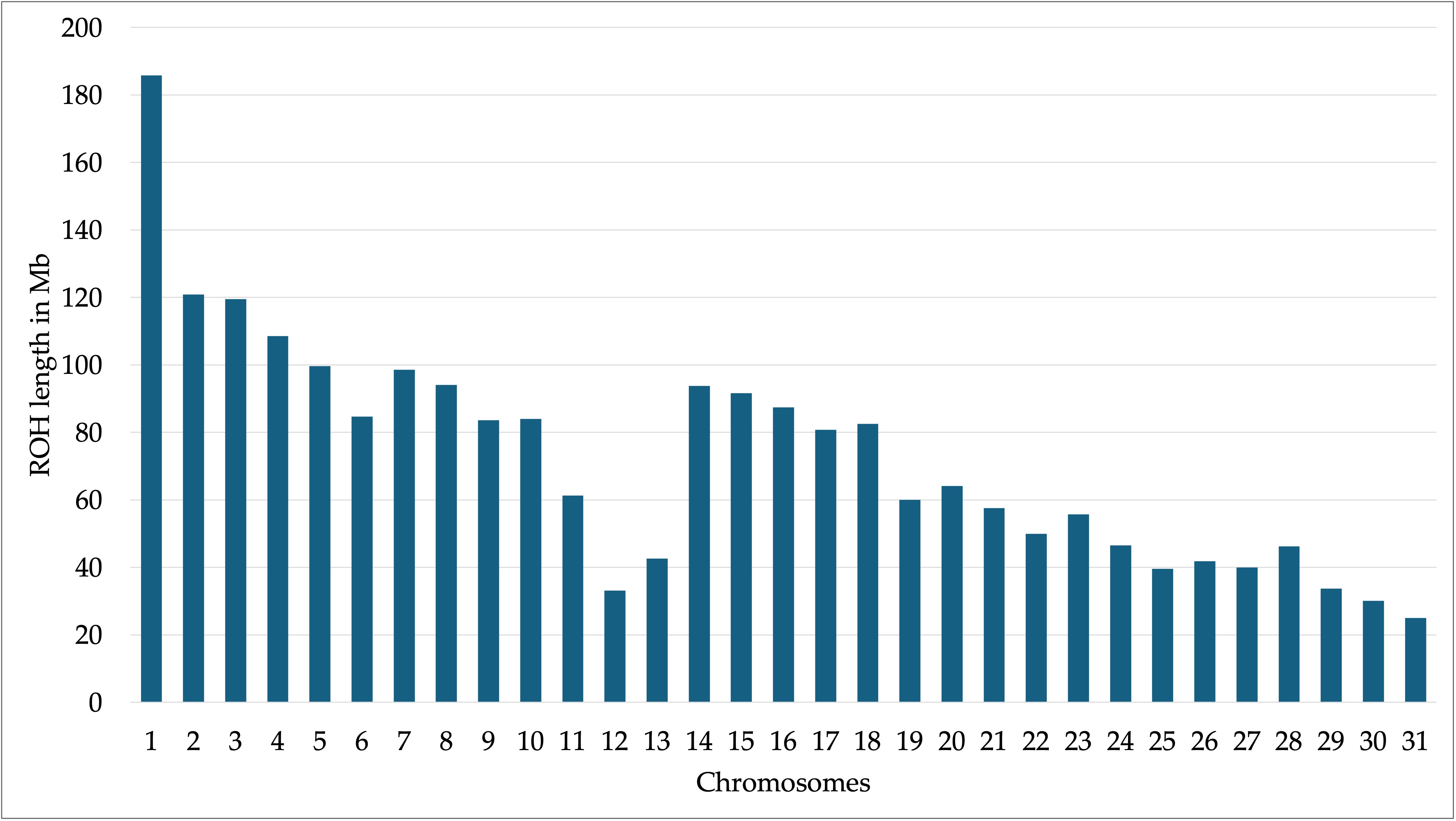

Supplement: Supplementary file 1 [file genes-16-00327-s001.zip › Figure S1 ROH lenght per chromosome.jpg]

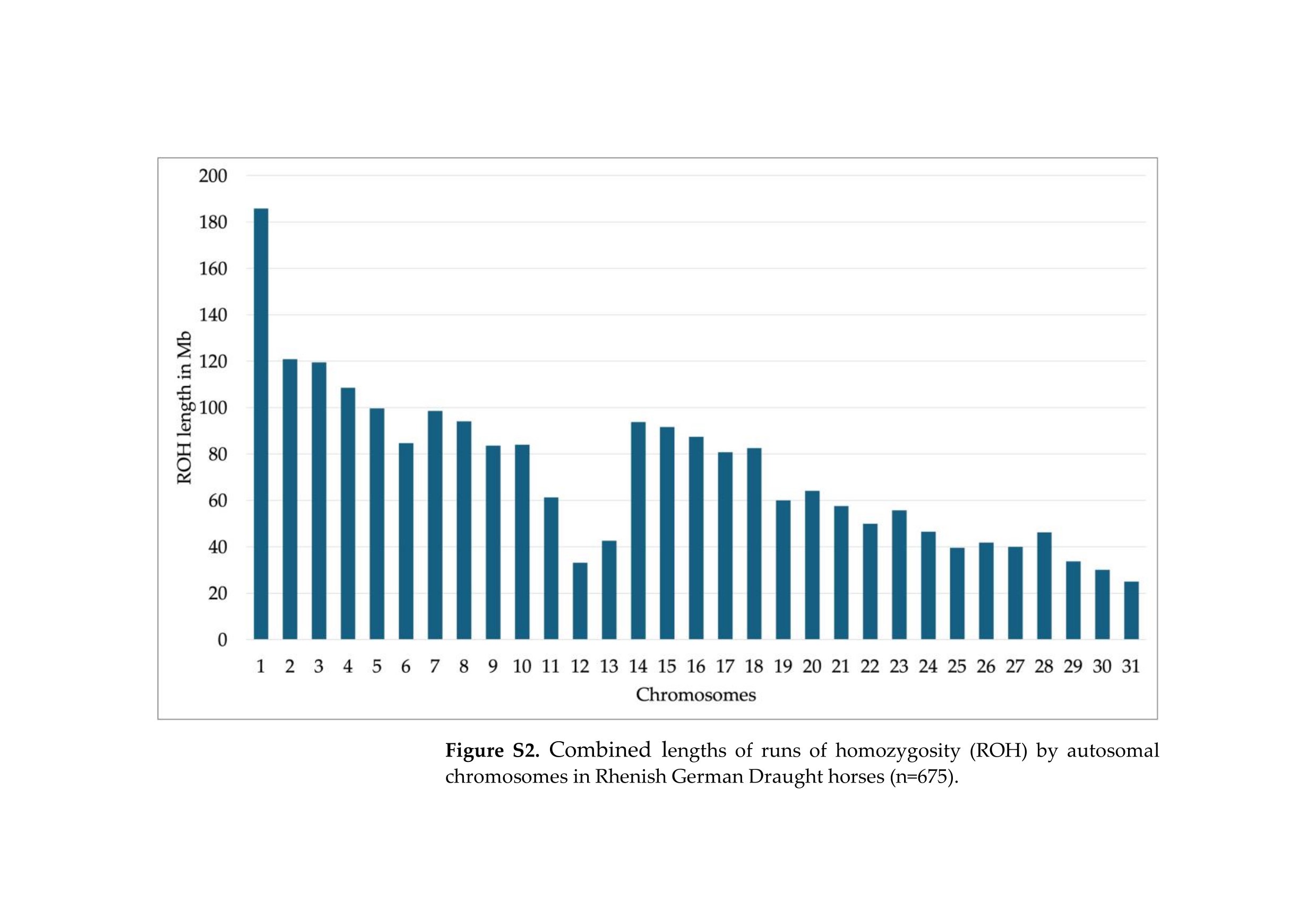

Supplement: Supplementary file 1 [file genes-16-00327-s001.zip › Figure S2. Length of ROH in Rhenish German Draught horses.jpg]
